# Life Trajectories, Biomedical Evidence, and Lessons for Policies

**DOI:** 10.3389/fpubh.2020.00160

**Published:** 2020-05-12

**Authors:** Paolo Vineis

**Affiliations:** ^1^Imperial College London, London, United Kingdom; ^2^Department of Computation & Data Science, Italian Institute of Technology, Genoa, Italy

**Keywords:** microsimulations, biomarkers, policies, biological capital, life-course

## Abstract

Here I compare two types of evidence that have recently emerged from the literature. This Commentary is a contribution to the Frontiers Research Topic on social disparities in aging, and aims to draw attention to the novel connections that link social disparities, the biological capital of individuals, and policy strategies. The biological capital (as defined in the paper), accrued since conception by individuals, in turn affects their social, cultural, and economic capitals, and thus creates a positive feedback loop. In a large network funded by the European Commission, Lifepath, we have shown that the determinants of health inequalities start in early life and cumulate throughout the life-course. For example, exposure to adverse childhood experiences (ACEs) influences the likelihood of later in life health effects, including poor aging. In this paper I compare two types of evidence that have recently emerged from the literature. One addresses the role of early vs. late exposures to risk factors for aging and mortality, including ACEs, using e.g., microsimulation models. The second type of evidence, provided in a recent document of the WHO European Regional Office, is based on the analysis of five broad determinants of health inequalities and eight different macroeconomic policies to tackle such inequalities. Six of the policies, if enacted, have the potential to reduce inequalities in the short term by increasing public expenditure on housing and community amenities, increasing expenditure on labor market policies, reducing income inequality, increasing social protection expenditure, reducing unemployment, and/or reducing out-of-pocket payments for health. Both of these lines of evidence suggest that there are ample opportunities for policy interventions. I also discuss the need for analytical methods to bridge the two types of analyses (biomedical and macroeconomic), i.e., fill the gap between analyses based on individual determinants of health inequalities and those based on societal determinants, to help create more effective policy-making. Also, I propose that before launching large projects to reduce health inequalities, well-designed experiments must be conducted to test their efficacy. These experiments, though, are challenging when addressing social policies, in consideration of ethical constraints and timescales.

## Introduction

The problem of inequalities in health has been extensively discussed in recent years after the publication of a few key scholarly contributions. The book by Piketty on the XXI century capital has drawn the attention of the world to rising inequalities (1), while the seminal paper by Case and Deaton included an unexpected phenomenon, i.e., increasing mortality (decreasing life expectancy) in the poor white population of the United States (2). Life expectancy has always and steadily increased after WWII, with the notable exception of Russia and neighbor countries after the collapse of the USSR. Case and Deaton's paper has attracted much attention and has led to similar analyses by the Lifepath consortium in Europe (described below) (3, 4), showing that in this continent there has been no decline in life expectancy after the recession, and in fact life expectancy has continued to rise, including in Eastern European countries. Average life expectancy in the WHO European Region increased from 76.7 years in 2010 to 77.8 years in 2015, though this trend obscures huge within-country differences (of course these estimates need to be revised after the dramatic COVID-19 epidemic). The conclusion of the authors of the Lifepath paper (3) is that, as opposed to the United States, the Welfare State in Europe (including its Eastern countries) acts as a buffer against the economic crisis and in general against the health decline of sectors of the underserved population. However, this conclusion needs to be verified and supported by further evidence, particularly given recent trends in the Welfare State in Europe. Questions that arise from recent research include: (a) what are the best entry points to attenuate health disparities in the life-course? (b) What is the best combination of individual health promotion and macroeconomic interventions by the State? (c) How can we complement observational and experimental evidence?

In recent years, new compelling evidence has emerged from original scientific research. Here I report some of the conclusions (a) of a large network on social inequalities in health, the EU-funded Lifepath initiative (4), and (b) of a document from WHO that suggests relevant policy directions. Lifepath was a Horizon-2020 consortium that brought together a large number of research teams in Europe and addressed health inequalities from the viewpojnt of intermediate mechanisms, including biomarkers, that lead from social inequalities to health impairment. There is a striking difference between the two approaches I summarize: the first follows the deeply rooted tendency to address individual-level determinants of health inequalities, based e.g., on apportioning the burden of disease to different factors including behavioral factors. The second approach is based on a method called decomposition, that refers to categories of distal determinants (i.e., at the societal level) of health inequalities.

In Lifepath we addressed what we called “biological capital.” Natural sciences focus in particular on biological mechanisms and outcomes, i.e., they address “*zoe*” (biological life), while social sciences address “*bios*” (biographical life). Epidemiologists aim to connect *zoe* and *bios*, though this attempt has rarely been explicit. The interplay of social contexts, behaviors and biomarkers in a life-course approach, like in Lifepath, is at the basis of biological capital that people accrue at any time point in their life trajectory.

This paper is not a systematic review, that would be very complex and would require the joint skills of different disciplines. My aim is to draw attention to the gap between the two types of evidence and suggests ways to fill it.

## Microsimulations as a Way to Address Policies

The first question I address is about the best entry points for interventions to attenuate health disparities at the individual level. An original piece of work provided within the Lifepath consortium deals with counterfactual models based on microsimulations from real data (5). Microsimulations are a set of tools that help address policy choices by modeling the impact of different measures, based on longitudinal cohorts. The authors of this analysis aimed to assess the potential impact of interventions targeting childhood and early life [reducing adverse childhood experience (ACE), and improving educational attainment], compared to the effect of interventions targeting adult life (reducing smoking). They used data from the 1958 NCDS British birth cohort and applied microsimulation models to estimate the expected mortality between age 16 and 55, under counterfactual assumptions that would decrease by half the observed level of exposure to (i) ACE, (ii) low educational attainment (at age 22), (iii) ACE and low educational attainment (a combined intervention), (iv) smoking at age 33. Results overall clearly stress the potential value of early interventions targeting ACEs and education, compared to well-recognized interventions on smoking (Table 1). This and other original research contributions from Lifepath have led to some general policy conclusions reported in the next paragraph. It is also worth noting that if ACEs were reduced and education increased individuals would be less likely to take up risky behaviors such as smoking. Policy-makers should consider prioritizing investment in early interventions, i.e., for children and families, and that would lead to less need for later interventions to address behavioral risks.

**Table 1:** Proportion of inequalities in mortality attributable to different factors amenable to intervention.

	**Impact on mortality at age 55**	
	**Women**	**Men**
Decrease by half of ACEs and low educational attainment in early life	−7.19% (−12.2 to 1.2)	−9.95% (−15.6 to 6.2)
Decrease by half of smoking at age 33	−7.10% (−13.1 to 1.1)	−12.29% (−16.9 to −5.91)

*Impact on total mortality at age 55 (5). ACEs, adverse childhood experiences*.

## Lifepath: The Right Interventions at the Right Time in a Life-Course Perspective

Microsimulation models are a scientific approach to answer our first question, i.e., in principle to identify the best entry points for action in a life-course perspective. In general, Lifepath research, mainly based on population studies (large cohorts) highlighted the importance of addressing early life inequalities to magnify the benefits of interventions to mitigate social disparities in health, including in adulthood and late life. Research showed the need to intervene on both traditional risk factors—such as smoking, alcohol, diet, BMI, physical activity—and on the factors that drive social deprivation starting in childhood.

According to Lifepath analyses, each stage of life requires different interventions that take into account context and timing. The biological consequences of early exposure to social disadvantage begin well before a person has fully taken up individual health behaviors like smoking or poor diet (children though may be exposed to passive smoking and poor diet from the outset). In particular, Lifepath outcomes point toward the major problem of the obesogenic and pro-inflammatory environments in early life and the need for primary prevention. Evidence suggests that expenditure and investment in a child's early years could be more effective and cheaper than later mitigation.

According to Lifepath analyses, young adults with disadvantaged social characteristics already show a higher biological risk compared to their more advantaged peers. The pre-existing biological risk is further exacerbated by the uptake of unhealthy behaviors. Lifepath has shown that living in deprived neighborhoods is associated with differences in health risks across the life-course, including detrimental lifestyle factors from childhood and adolescence onwards, and worse glucose metabolism from early adulthood (6). The effects of early life social disadvantage on biology may amplify from early adulthood by age 25 (6). Addressing social exposures and health behaviors early in adulthood can limit their long-term effects and mitigate amplifications. Overall, the accumulation of biological fingerprints of adverse conditions and hazardous exposures has an effect on the individual's *biological capital*, a concept that adds to the well-known concepts of economic, social and cultural capitals as proposed long ago by Bourdieu (7).

Based on Lifepath studies, we know that by mid- and late-adulthood premature mortality disproportionately affects socially disadvantaged people, and that social patterning in physical functioning, physiological wear-and-tear, and in molecular processes including epigenetic age acceleration is observed (4). All these changes are also mediated by risk factors including in particular smoking, BMI and metabolic disorders, such as fatty liver disease and diabetes. In this phase of life we are interested in a *harm reduction* approach, i.e., mitigating the risks of previous exposures. This implies both primary prevention to avoid or interrupt exposure to hazardous behaviors and environments, and early identification of health damage through appropriate detection tools.

As Bourdieu has suggested in his sociological contributions, and Lifepath has reinforced from a biomedical viewpoint, the overall effect of social disadvantage comes from an interplay of individual and contextual factors.

An important chapter of educational investments in childhood is health education. On top of the positive effect of education in general (through mechanisms such as neural development and biological aging), there is a specific role for early health education, tackling health literacy, health behaviors, sense of control and empowerment (8). According to a review, the best indicator for the success of educational systems in improving health of children and adolescents (and prevent risky behaviors) is the quality of schooling at all levels (8).

## The HESR Who Exercise

How can we complement interventions at the individual level, as mainly done in Lifepath's models, and the macroeconomic choices? Further, what is the evidence supporting the effectiveness of the latter? An important and recent source of information is the “European Health Equity Status Report” of the European Office of WHO. This report includes estimates of the effect of general explanatory factors on health (measured as self-reported health status, mental health and life satisfaction) (9). Data from several sources were used to derive the indicators featured in the Health Equity Dataset. Data were obtained from sources such as household and other population-based surveys, administrative data systems and surveillance systems. As Table 2 shows, the greatest contribution is associated with Income Security and Social Protection (explaining 35% of inequalities in self-reported health), followed by Living Conditions (29%), and Social and Human Capital (19%). They have also considered the potential effects of eight *macroeconomic policies* in reducing health inequities. The improvement has been measured by the percentage reduction in limiting illness reported among adults in the highest and lowest income quintiles within countries. Increasing per-capita income was the only policy which showed no association with reducing health inequalities. Six of the policies had statistically significant potential to reduce inequalities in illness among adults in the short term: increasing public expenditure on housing and community amenities; increasing expenditure on labor market policies; reducing income inequality; increasing social protection expenditure; reducing unemployment; and reducing out-of-pocket payments for health (9).

**Table 2:** WHO HESR: five conditions' contributions to inequalities in self-reported health (EU countries) (9).

Health Services	10%
Income Security and Social Protection	35%
Living Conditions	29%
Social and Human Capital	19%
Employment and Working Conditions	7%

It is worth giving some details for some of the distal (i.e., more distant from the single individual's choices and behaviors) societal determinants, that shed light on potential entry points for macroeconomic policies. Between 35 and 46% of the health inequalities in self-reported health, mental health, and life satisfaction were associated with income insecurity and lack of social protection. It is concerning that the overall trend across the WHO European Region shows declining income security among people who are least well-off. According to the report, the aim of social protection policies is to help ensure that all people have access to income security throughout their lives, through: access to essential health care, including maternity care; basic income security for children, providing access to nutrition, education, care and any other necessary goods and services; basic income security for people of working age who are unable to earn sufficient income; and basic income security for older people.

According to the report, on average, 17 of every 100 people live in relative poverty across the Region, an increase from 15 of every 100 in the year 2005 (defined as the percentage of people living on or below 60% of median household disposable income after taxes and transfers). Living and housing conditions also play a major role in health inequalities: in particular, over 70% of the gap in health inequalities in self-reported health status linked to living conditions can be explained by differences in housing and fuel deprivation.

Between 7 and 19% of the health inequalities in self-reported health, mental health, and life satisfaction are associated with social and human capital, and over two thirds of this contribution are explained by educational differences. The importance of education is stressed by its inter-generational effect: among parents with a high level of education, a much smaller proportion of their children end up having just primary-level education, compared to parents with lower education.

## A Conceptual Conundrum: What are the Pathways to Health Inequalities?

As we have seen, the topic of the determinants of health inequalities can be addressed from different perspectives. One common approach involves investigating what we call “proximal” determinants, i.e., risk factors of disease that are mainly related to the individual behavior or immediate living environment (including ACEs). The microsimulation approach described above is probably the most innovative to investigate life-course inequalities at the individual level. The other approach I have described (WHO HESR) is based instead on distal determinants, i.e., social factors that are more distant from the individual and are overarching. In fact, the problem with these approaches, and that is at the basis of several statistical misunderstandings, is that the two should be treated as complementary. Smoking, for example, is a proximal behavioral cause of disease, but it is also unevenly distributed across socio-economic strata, so that part of the effect of low social conditions is indirect, i.e., mediated by smoking. Another component of the effect of social conditions is instead direct, i.e., due to other (including unknown) factors. Obviously the distinction between distal and proximal is blurred, since poor housing, for example, belongs to both.

The interaction of the two approaches has been seldom reported in the published literature, and the way ahead is based on more complex statistical analyses that should be able to take into account the multi-layered nature of the problem, for example using mediation analysis. An example is a study based on seven cohort studies participating in the Lifepath consortium (total n = 179,090) (10). Using both socioeconomic position (SEP)(based on occupation) and education, authors estimated the direct effect of SEP on all-cause mortality, and the indirect effect via the joint mediating role of smoking, alcohol intake, dietary patterns, physical activity, body mass index, hypertension, diabetes, and coronary artery disease. Mortality was reduced via modeled hypothetical actions of increasing SEP or education. Through higher education the risk of death directly due to SEP was 14% lower for women and 29% lower for men, compared to lower education. On top of this, 45% (for women) and 38% (for men) of the SEP effect was jointly mediated by the intermediate risk factors. These observational findings support policies to reduce mortality both through improving socioeconomic circumstances (including increased education), and by altering intermediaries, such as lifestyle behaviors and morbidities. This paper is one of the very first to adopt a mediation analysis approach in this field, i.e., to consider upstream socio-economic conditions and downstream risk factors to explain health outcomes (10). However, the mediation analysis approach requires a further step forward, i.e., the ability to incorporate also overarching contextual determinants that precede individual social and behavioral determinants. An example of joint analysis of individual and contextual factors has been published from four cohorts (11). Participants living in the most deprived quintile had 1.13 times higher allostatic load, i.e., neighborhood deprivation was associated with biological wear and tear, suggesting that neighborhood-level interventions may yield measurable individual health gains (11). A further step forward would be to extend this kind of analyses also to the impact of different policy choices.

## What are the Best Policy Approaches?

A final question is how to best complement observational research with experimental approaches. Unfortunately, researchers have provided limited evidence so far about the most effective policies to be implemented to mitigate the health effect of social disadvantage. Whereas, there is consent on the fact that the shrinkage of State policies in support of the most disadvantaged is deleterious, the most effective and efficient policies are under scrutiny. Economists and policy-makers have proposed different potential approaches, such as conditional cash transfer (CCT), differential taxation models, proportionate universalism, etc. It is clear that one size does not fit all. For example, CCTs were effective in Central and South America in reducing the health effect of social disadvantage, but less so in New York (4). There is no systematic comparison across the different alternative policies, and randomized controlled trials are very few. To my knowledge no systematic review comparing alternative approaches has been published so far. In spite of the knowledge gaps, the WHO document clearly shows that—based on what is known—much could and should be done immediately to attenuate health inequalities. It is imperative to consider the *biological capital* as one of the main resources individuals have, not only to improve their health status and quality of life, but also to increase their share of economic, social, and cultural capital (12).

## Author Contributions

The author confirms being the sole contributor of this work and has approved it for publication.

## Conflict of Interest

The author declares that the research was conducted in the absence of any commercial or financial relationships that could be construed as a potential conflict of interest.

## References

[1] Piketty T. Capital in the Twenty-First Century. Cambridge, MA: Harvard University Press (2014). 10.4159/9780674369542

[2] Case A Deaton A. Rising morbidity and mortality in midlife among white non-Hispanic Americans in the 21st century. Proc Natl Acad Sci USA. (2015) 112:15078–83. 10.1073/pnas.1518393112 26575631 PMC4679063

[3] Mackenbach JP Valverde JR Artnik B Bopp M Brønnum-Hansen H Deboosere P. Trends in health inequalities in 27 European countries. Proc Natl Acad Sci USA. (2018) 115:6440–5. 10.1073/pnas.1800028115 29866829 PMC6016814

[4] Vineis P Avendano-Pabon M Barros H Chadeau-Hyam M Costa G Dijmarescu M. The biology of inequalities in health: the Lifepath consortium. Front Public Health. 10.3389/fpubh.2020.00118 PMC7235337 32478023

[5] Lepage B Colineaux H Kelly-Irving M Vineis P Delpierre C Lang T. Simulating counterfactual public health interventions on social factors in early life or on smoking in adulthood - results from the 1958 NCDS British birth cohort. (in press).

[6] Kivimäki M Vahtera J Tabák AG Halonen JI Vineis P Pentti J. Neighbourhood socioeconomic disadvantage, risk factors, and diabetes from childhood to middle age in the Young Finns Study: a cohort study. Lancet Public Health. (2018) 3:e365–73. 10.1016/S2468-2667(18)30111-7 30030110 PMC6079015

[7] Bourdieu P. The forms of capital. In: Richardson J, editor. Handbook of Theory and Research for the Sociology of Education. New York, NY: Greenwood (1986). p. 241–58.

[8] Cohen AK Syme SL. Education: a missed opportunity for public health intervention. Am J Public Health. (2013) 103:997–1001. 10.2105/AJPH.2012.300993 23597373 PMC3698749

[9] Healthy Prosperous Lives for All: The European Health Equity Status Report. Copenhagen: WHO Regional Office for Europe (2019).12216768

[10] Laine JE Baltar VT Stringini S Gandini M Chadeau-Hyam M Kivimaki M. Reducing socio-economic inequalities in all-cause mortality: a counterfactual mediation approach. Int J Epidemiol. (2019) dyz248. 10.1093/ije/dyaa008. [Epub ahead of print]. PMC7266549 31855265

[11] Ribeiro AI Fraga S Kelly-Irving M Delpierre C Stringhini S Kivimaki M. Neighbourhood socioeconomic deprivation and allostatic load: a multi-cohort study. Sci Rep. (2019) 9:8790. 10.1038/s41598-019-45432-4 31217447 PMC6584573

[12] Vineis P Kelly-Irving M. Biography and biological capital. Eur J Epidemiol. (2019) 34:979–82. 10.1007/s10654-019-00539-w 31342230

